# Influence of the femoral offset on the muscles passive resistance in total hip arthroplasty

**DOI:** 10.1371/journal.pone.0250397

**Published:** 2021-05-04

**Authors:** Stanisław Burzyński, Agnieszka Sabik, Wojciech Witkowski, Piotr Łuczkiewicz

**Affiliations:** 1 Department of Mechanics of Materials and Structures, Faculty of Civil and Environmental Engineering, Gdańsk University of Technology, Gdańsk, Poland; 2 II Clinic of Orthopaedics and Kinetic Organ Traumatology, Medical University of Gdansk, Gdańsk, Poland; Assiut University Faculty of Medicine, EGYPT

## Abstract

**Background:**

Soft tissue tension is treated as a crucial factor influencing the post-THA dislocation. The femoral offset is regarded as one of the major parameters responsible for the stabilization of the prosthesis. It is unclear which soft tissue is mostly affected by the offset changes.

**Methods:**

A finite element model of the hip was created. The model comprised muscles, bones, a stem, the acetabular component and a liner. The muscles were modelled as a Hill-type musculo-tendon nonlinear springs. Nonlinear analyses of the hip flexion and internal rotation were performed for the two values of the femoral stem offset.

**Results:**

We observed that the quadratus femoris and gluteus medius produce the largest resisting moment opposing the external load excreted by the surgeon during the intraoperative hip dislocation test.

**Conclusions:**

An increased femoral offset increases the stretching of the quadratus femoris muscle significantly and provides the growth of its initial passive force. This muscle serves as a stiff band, providing stabilisation of the hip prosthesis, measured during the simulated intraoperative test.

## Introduction

Total hip arthroplasty (THA) is the most successful and cost-effective procedure for patients with end-stage hip arthritis [1]. Surgical complications are infrequent and include infection, aseptic loosening, fracture, leg length discrepancy and hip instability. Postoperative dislocation is the second most common complication of total hip arthroplasty after aseptic loosening [2].

The aetiology of instability can be divided into two categories: surgery-related factors and patient-related factors. Patient-related factors are associated with age, sex, height, weight, soft-tissue laxity, previous surgery and neuromuscular impairment [3]. Surgery-related factors include the following: surgical approach, component orientation, choice of implant, soft tissue repair, maintenance of the leg length and femoral offset [4–6]. One of the most important factors in preventing this complication is restoring the femoral offset, defined by the distance between the centre of the femoral head and a perpendicular line drawn through the centre of the stem [7]. Increasing the femoral offset has been shown to increase the range of motion, restore hip biomechanics, reduce the occurrence of impingement, increase the strength of the abductor muscles and maximize joint stability by tensioning the soft tissue [8, 9]. Although inappropriate soft tissue tension is cited as a crucial factor for post-THA dislocation, few investigations have addressed this problem. Most have focused on the abductor mechanism neglecting the role of other muscles contributing to hip stability [10–12]. The kinetic factors that govern whether the implant will dislocate are beyond the scope of clinical study. In particular, the influence of soft tissue, which contributes to creating the resisting moment, has been an under-developed area of clinical investigation [13, 14]. Thus, finite element method (FEM) models [15, 16] were presented, providing the study of the abductor muscle contribution to total hip prosthesis stability.

In this study, we used the FEM model to analyse the effect of the femoral offset on the passive tensile reactions of muscles crossing the hip joint in the posterior approach arthroplasty. We did not limit the study to the abductors because the stability of the joint is assumed to also be afforded by other, particularly short, muscles [12]. The motivation for this simulation is the observable influence of the femoral offset on preventing dislocation [17].

## Materials and methods

### Geometry

One finite element method (FEM) model of the pelvis, femur and hip joint was created together with the respective muscles and tendons. The geometry of the pelvis and femur was obtained from the project “*ViVA—Virtual Vehicle Safety Assessment*: *Open Source Human Body Models addressing gender diversity*” (https://www.chalmers.se/en/projects/pages/openhbm.aspx). Specifically, the results described in [18] were used. The pelvis and femur finite element (FE) mesh was transferred to Hypermesh (Altair Engineering, Troy, Michigan, USA) and orientated to the supine position of the body. The centre point of the femur’s head was established as the centre of the sphere, fitting best the original geometry of the femur’s head. The original FE mesh was refined, and then the prepared model was transferred to the Abaqus v.6.14 (Dassault Systemes Simulia Corp., Providence, USA) FEM system where geometrically nonlinear analysis was performed, accounting for large rotations, displacements and the contact effect.

### Muscle modelling

The muscles and tendons are treated as springs whose response is described by the widely used Hill’s type musculo-tendon model [19–24]. Details of mathematical formulations are presented in S1 Appendix. The properties of the muscles are taken from [22, 25] where statistical data from cadavers is presented. In Abaqus, the musculo-tendons were introduced as *Connector Section, type *Axial with the following behaviour options: *Connector Elasticity, nonlinear, and *Connector Constitutive Reference (length). The values of forces as the function of length were calculated from the above data and formulae through Python scripts. The origin and insertion of each musculo-tendon were verified to conform to its anatomical line. The insertion points of the musculo-tendon attached to the tibia (not accounted for) were reconstructed and positioned in space based on the anatomical evidence.

**Fig 1:**
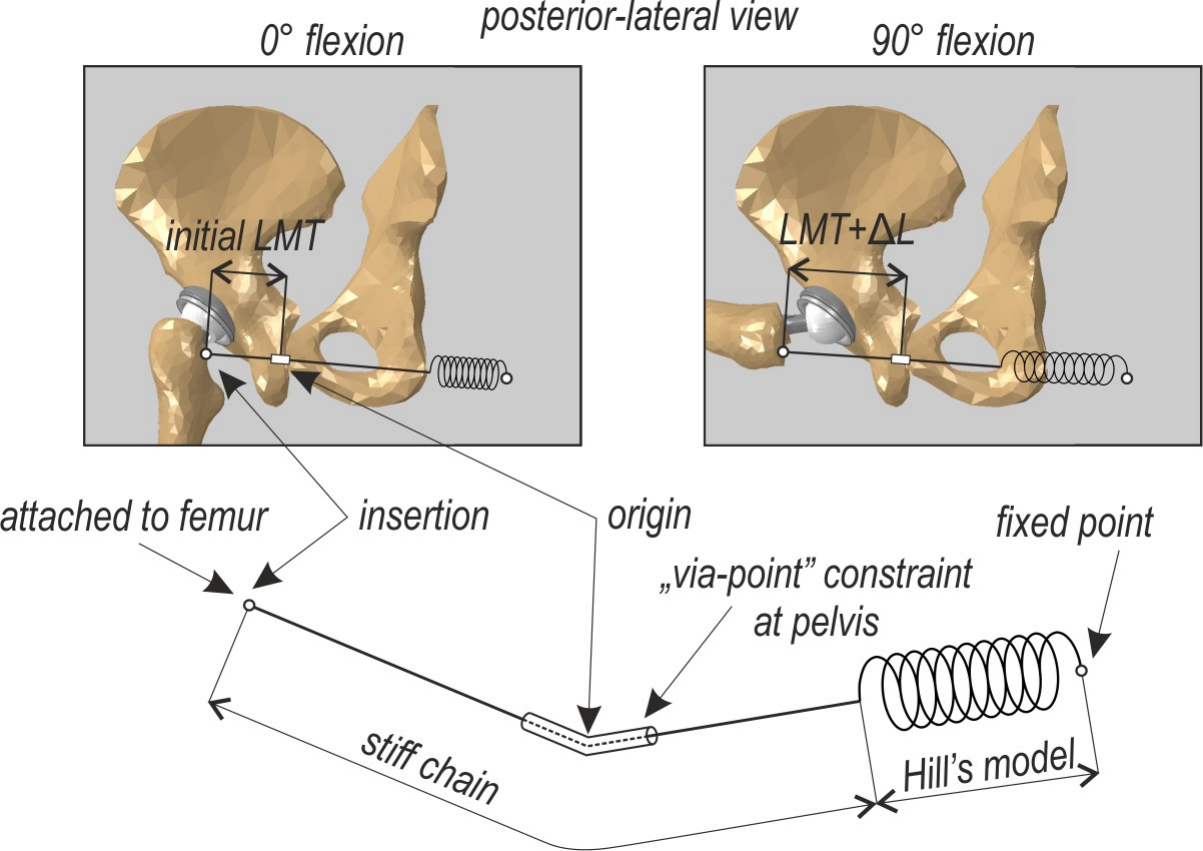
Example of muscle’s spatial modelling, here quadratus femoris. The same approach is used for all other muscles.

Fig 1 depicts, using the example of the quadratus femoris, the authors’ approach to model curvilinear muscle paths in Abaqus. Note that all the muscles are included in the FEM model based on this approach. The technique presented here relates closely to experimental setups used for measurements in cadavers—e.g. [26]. At insertion, we attached a rigid chain capable of transmitting tension force only. The chain is free to wrap around bones and artificial wrapping surfaces, compared with [22], thus preserving physiological lines of action of the muscles despite the lack of the 3-dimensional volume. Moreover, the chain passes the “via-point” constraint placed at the physiological origin. Next, the chain continues to the attachment point with a nonlinear spring with force displacement relation given the described Hill-type muscle model. Such an approach preserves the physiological line of action and proper calculation and transfer of muscle forces.

### Bones and implant

**Fig 2:**
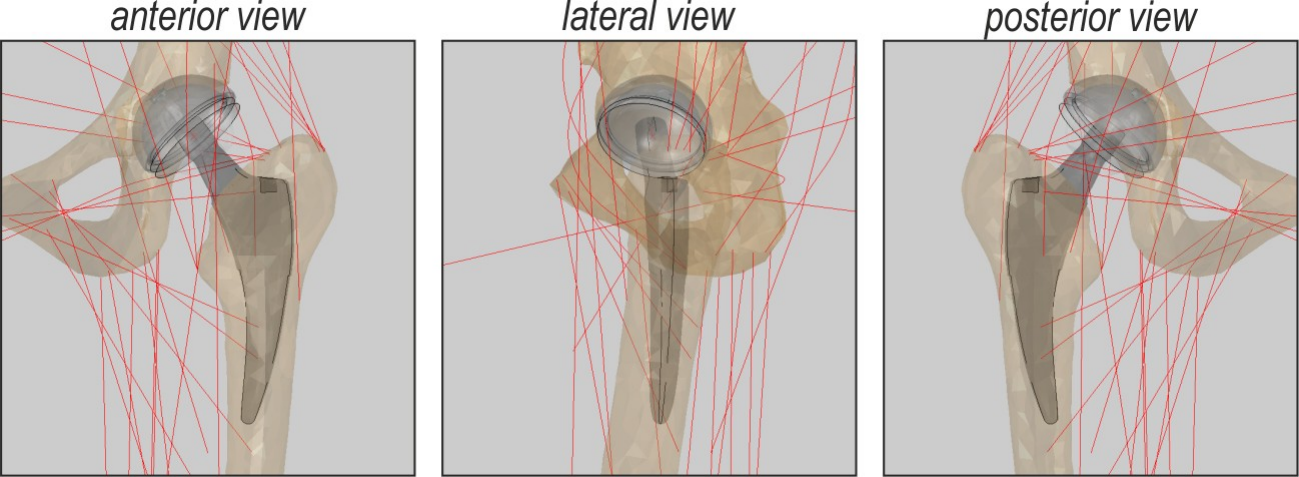
Stem position. The red lines denote the Hill-type muscle axes.

**Fig 3:**
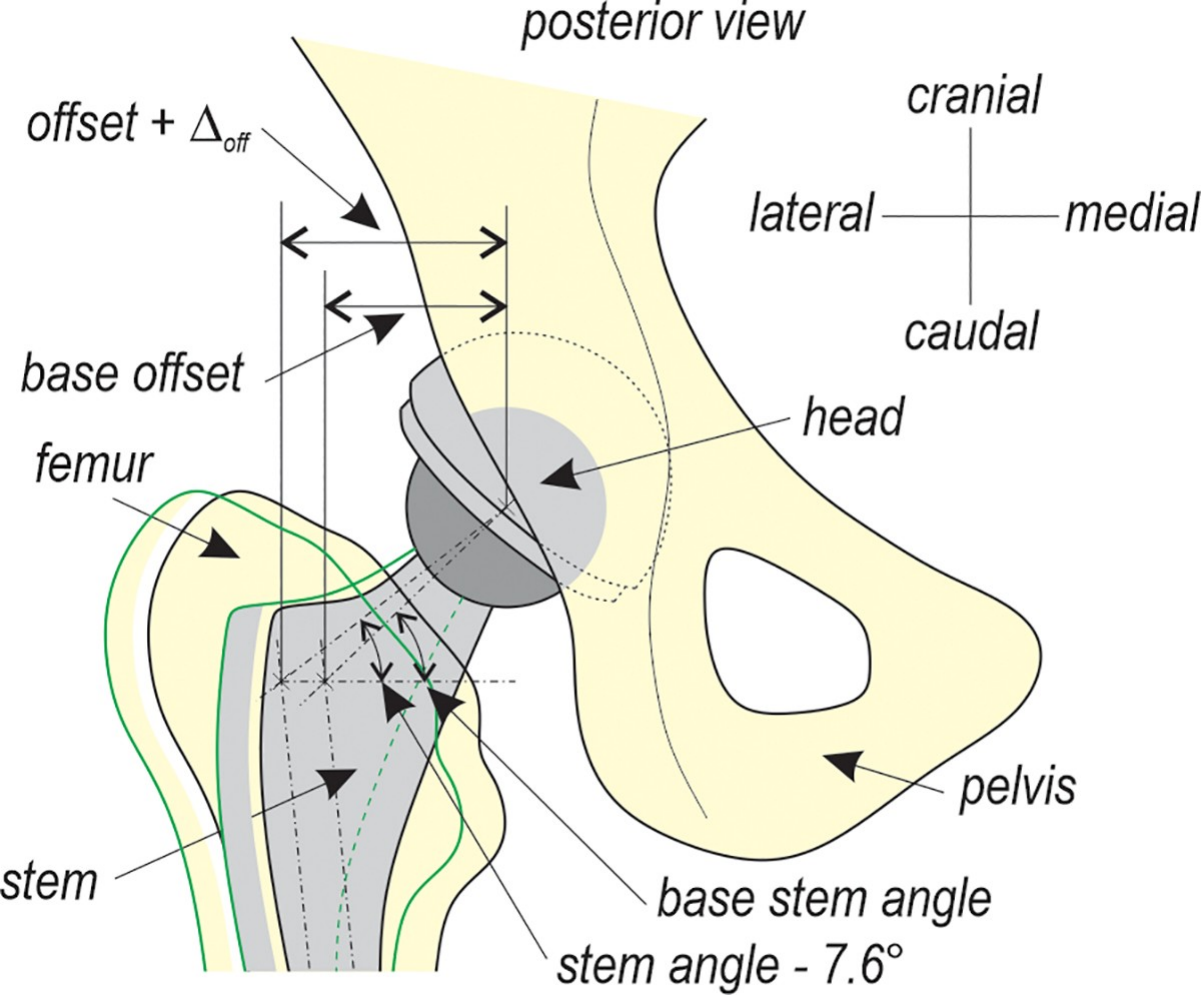
Offset definition. The green colour denotes a high offset.

The bones, stem and head acetabular component are assumed to be rigid. The liner was modelled as isotropic and linear elastic, assuming the following data: *E* = 850.0 GPa, Poisson ratio *v* = 0.4, and *ρ* = 960 kg/m^3^. The position of the implant relative to the femur and pelvis is shown in Fig 2. The cup was virtually implanted in the acetabulum so that it remains at the anatomical centre of rotation. The cup inclination angle and anteversion angle were 45°and 10°, respectively. Two stems of the same size, with different offsets, were placed parallel to the long axis of the femur with an anteversion angle of 10°, preserving the leg length The neutral position of the stem is understood as the position which does not affect the centre of rotation and the natural distance between the pelvis and the femur bone. The difference in the offset between the standard-offset stem and high-offset stem was 7 mm. The difference in the stem’s neck angle was 7,6°. Fig 3 shows the definition of the femoral offset as obtained from measurements of the commercially available product.

### Boundary conditions

Following [22], the main coordinate system is placed at the midpoint between the left and right anterior superior iliac spines. In the initial position, the *x*-axis points distally, the *y*-axis points laterally and the *z*-axis points anteriorly. All the rigid bodies described above are assumed as fixed in the simulation. The movement of the femur is controlled by the rotational velocity vector applied at the centre of the femoral head.

To examine the femoral offset influence on the hip stability and muscle tension, one of the practical intraoperative stability tests is simulated [27, 28]. In this test, designed to check the stability against posterior dislocation, the surgeon flexes the knee up to 90°, flexes the hip up to 90° and rotates it internally to check manually whether the soft tissue resistance is sufficient to provide prosthesis stability.

## Results

### Validation

**Fig 4:**
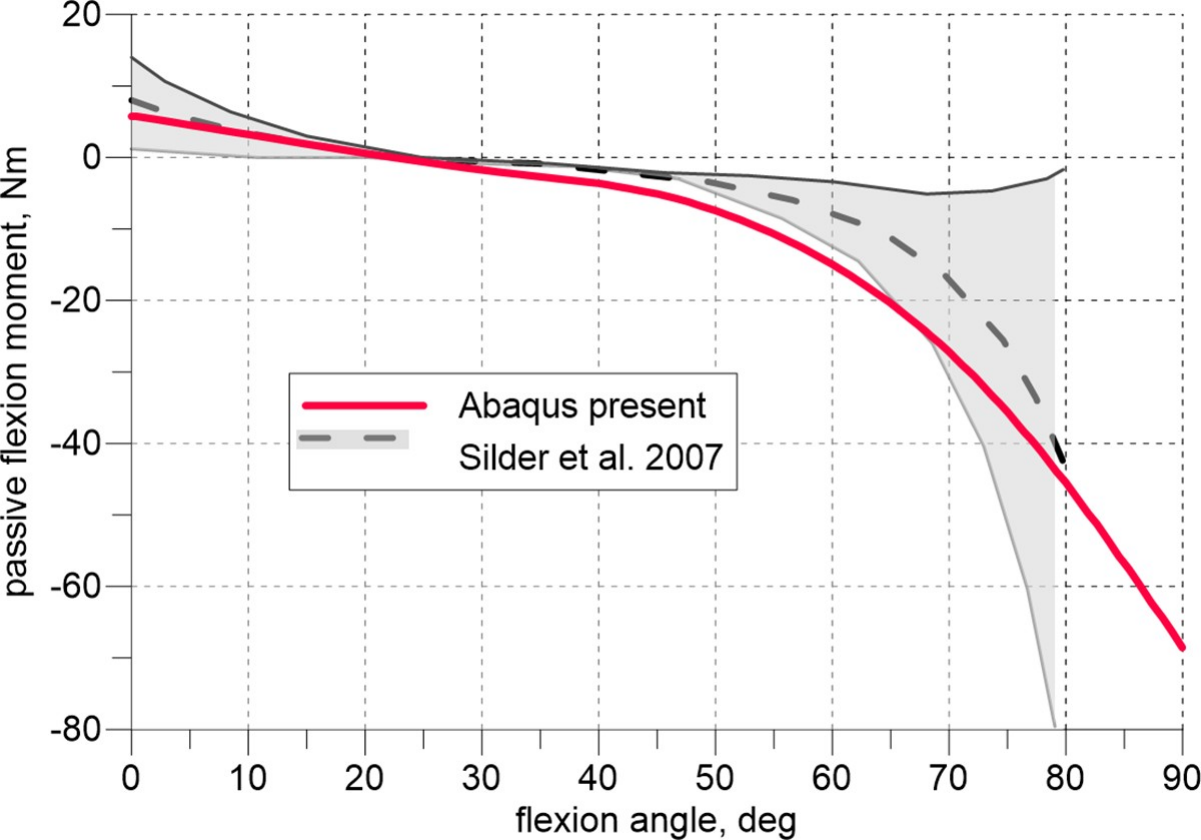
Passive hip flexion moment vs. flexion angle; 15° knee flexion.

The model was validated against the experimental data available in the literature. Fig 4 depicts the comparison of the flexion curve (passive moment vs. flexion angle) calculated with the present model to the experimental data reported in [29] (mean value and standard deviation). The experimental data were obtained there on twenty healthy young adult volunteers. The flexion of the hip joint was preceded by the 15° knee flexion. The moment is calculated relative to the centre of the femur head. The model predicts well the experimental results.

**Fig 5:**
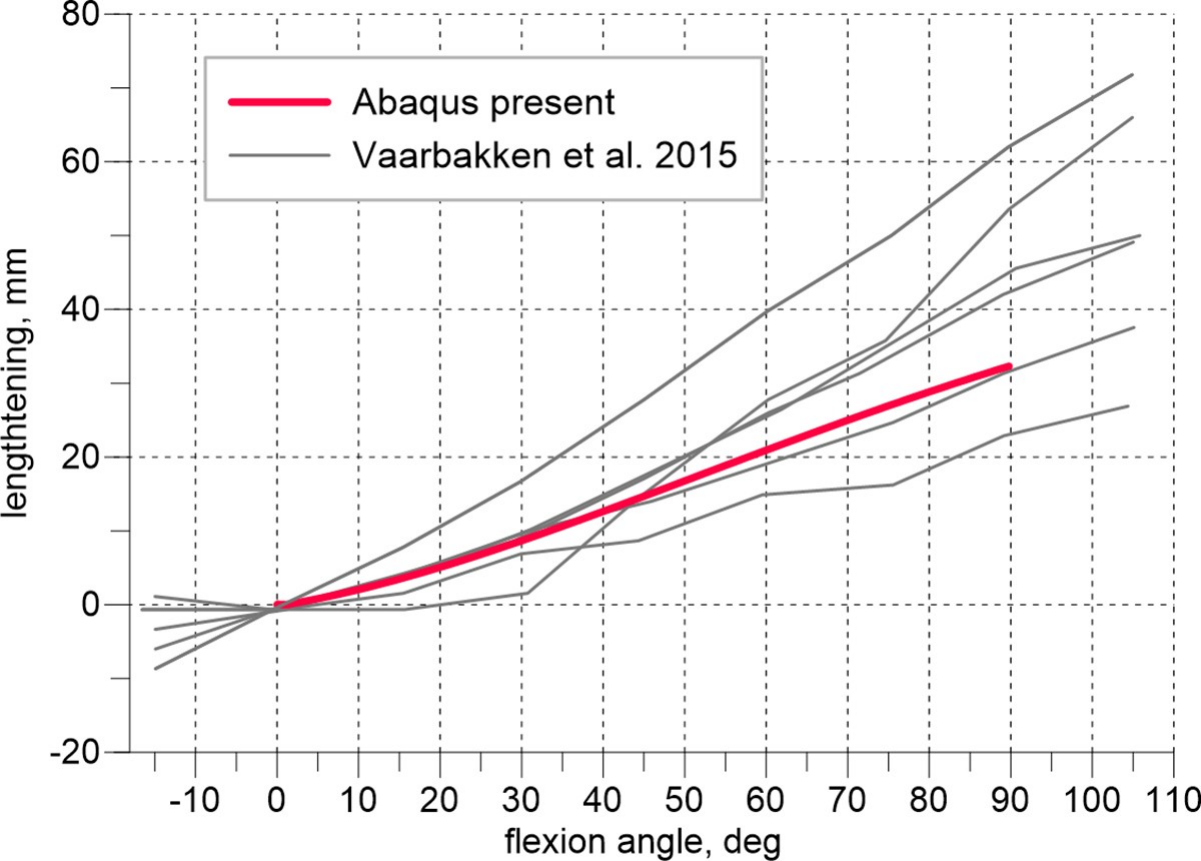
Lengthening of the quadratus femoris vs. hip flexion angle.

**Fig 6:**
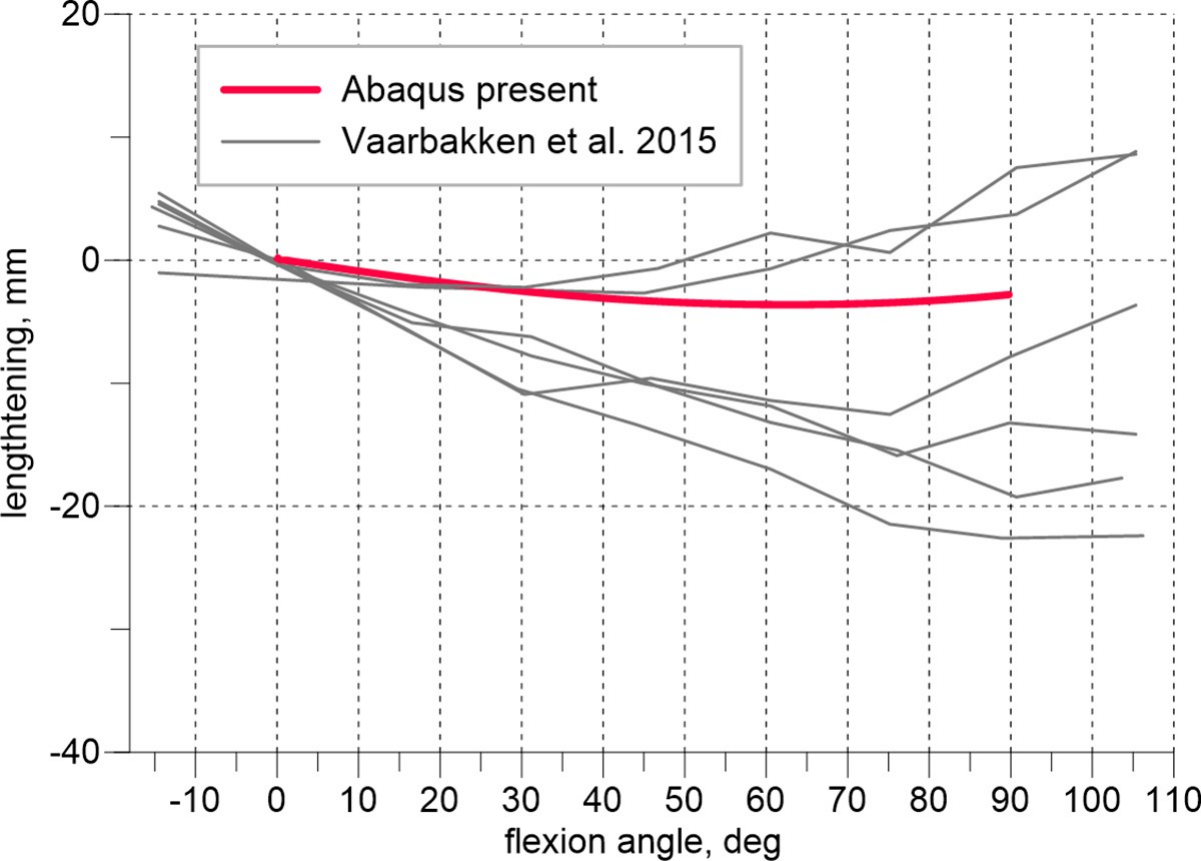
Lengthening of the obturator externus vs. hip flexion angle.

Figs 5 and 6 illustrate our results of the elongation of the quadratus femoris and obturator externus during the hip flexion compared with the data measured experimentally [26]. Six solid grey line curves refer to the experimental lengthening measurements of 2 muscle strings of 3 cadaveric hips. The results obtained using the present model fall within the range determined by the experiments.

### Stem offset vs dislocation

**Fig 7:**
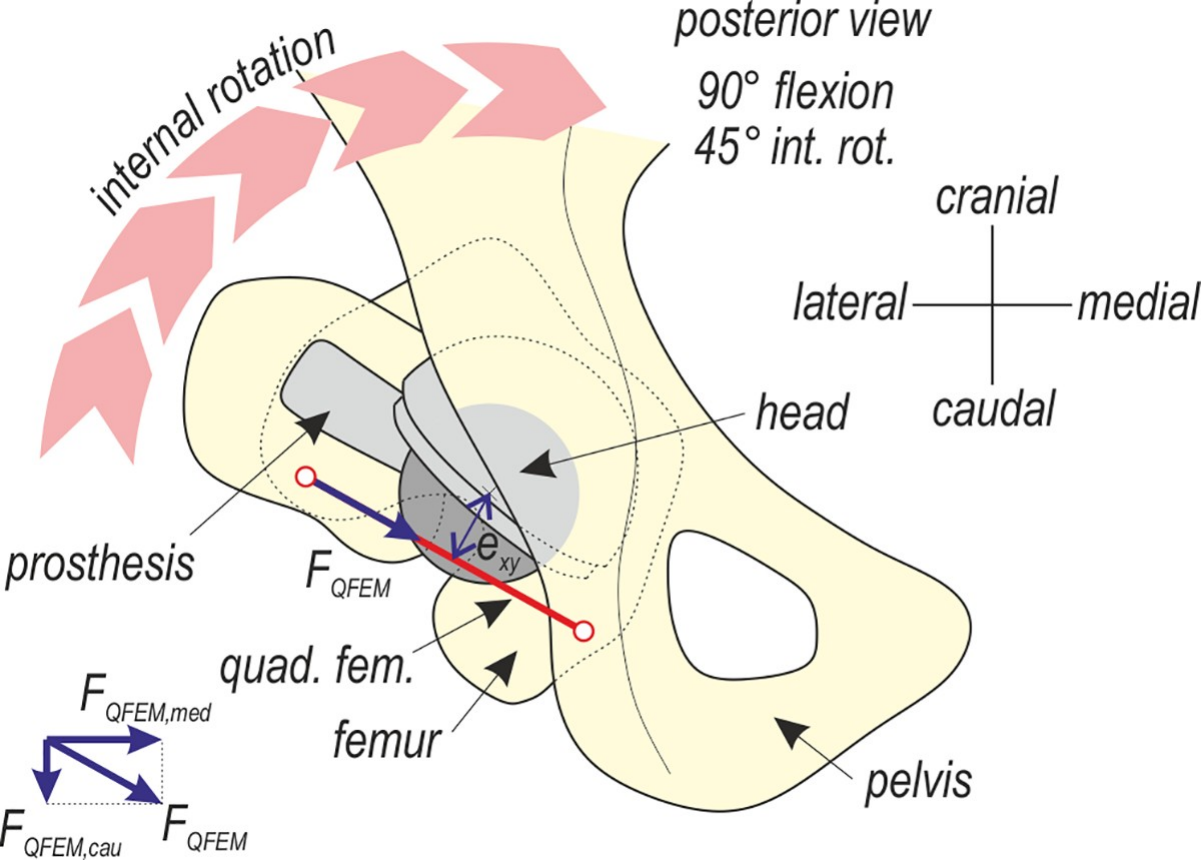
Posterior view of the quadratus femoris during the intraoperative manoeuvre.

**Fig 8:**
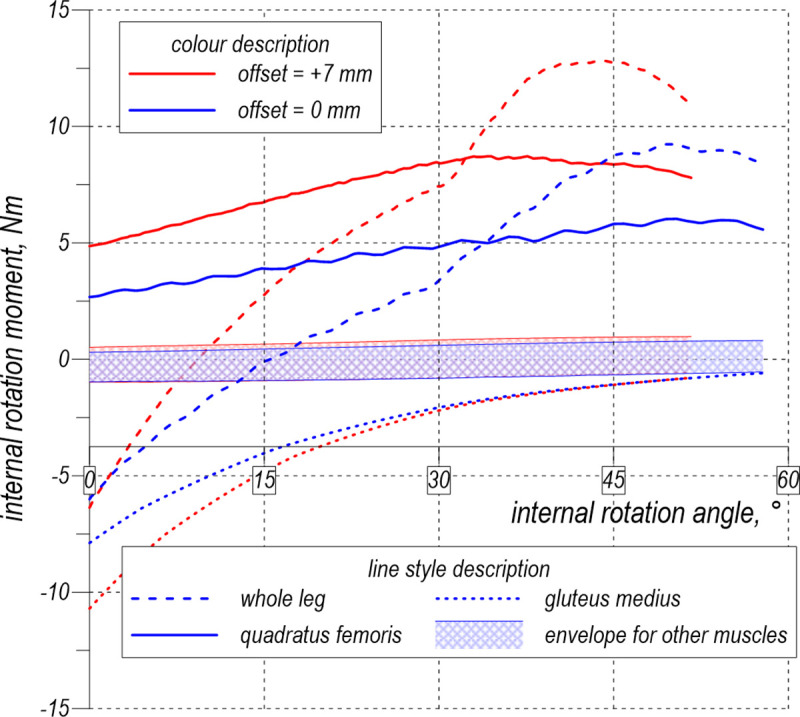
Analysis of the passive internal rotation moment during the intraoperative manoeuvre.

To assess the influence of the offset, the model of the hip in Abaqus was loaded by the rotational velocity first to achieve 90° flexion and then to simulate internal rotation (Fig 7). In the simulated posterior approach arthroplasty it was assumed that piriformis was cut and not included in the analysis. Two values of the offset were assumed: 0 mm and +7 mm. The passive internal rotation moments calculated by the model are shown in Fig 8. All the muscles except for the quadratus femoris and gluteus medius produced small moments of approximately±1 Nm. These values are collected together in Fig 8 as the envelope. Quadratus femoris and gluteus medius play predominant roles in resisting the internal rotation compared with the total passive moment from all the muscles. Regarding the total moment curves of the leg, characteristic load limit points can be observed corresponding to the maximum passive moments attained during the internal rotation. These moments depend on the offset value and are equal to 8.97 Nm and 12.70 Nm, see Fig 8.

**Fig 9:**
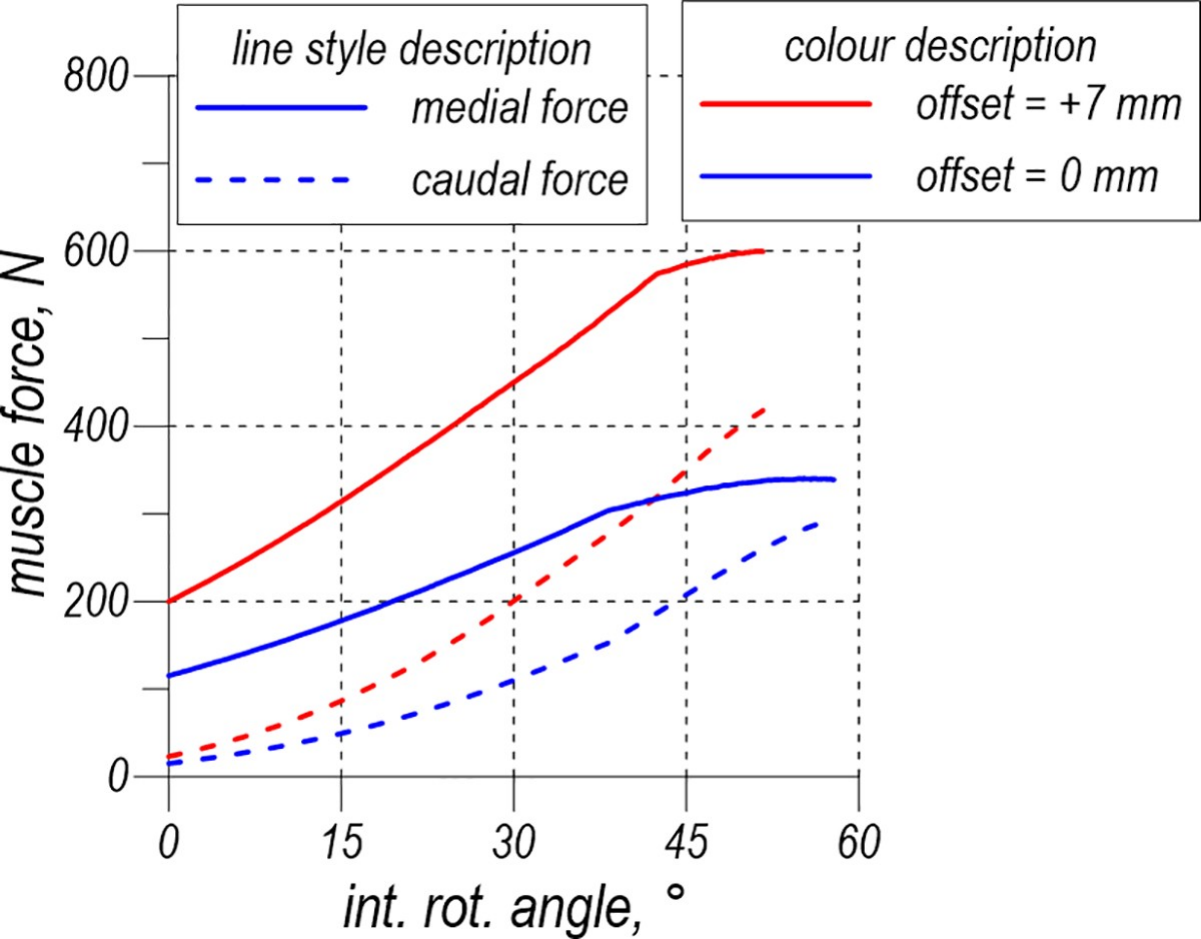
Force analysis for the quadratus femoris.

Fig 9 shows curves of the medial and caudal force components in the quadratus femoris versus internal rotation angle for the two studied offsets. These force components are very sensitive to the offset change.

**Fig 10:**
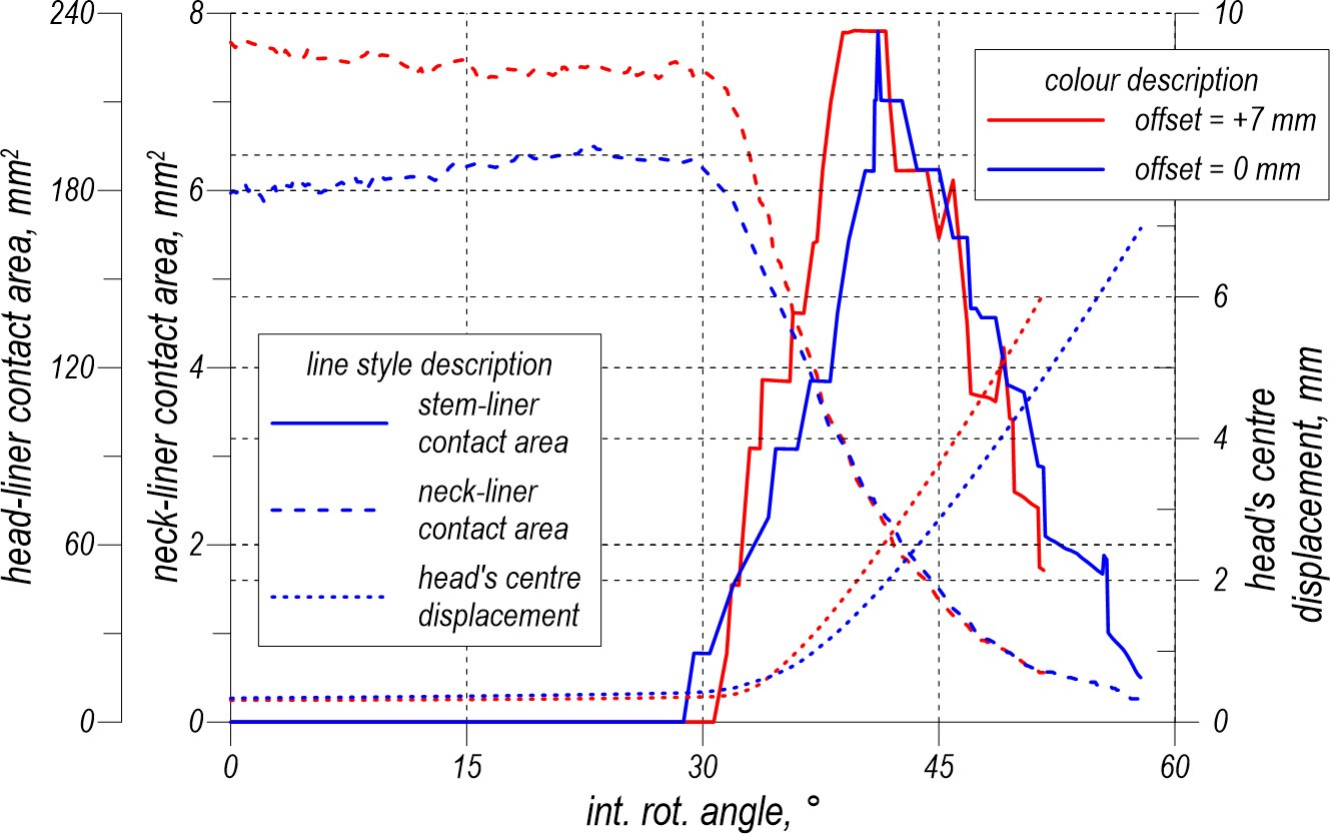
Analysis of the contact area and head centre displacement.

Fig 10 depicts the changes in the stem-liner and head-liner contact areas and the head’s centre displacement. The head displacement is the magnitude obtained from the three displacement components. In the initial phase of the rotation manoeuvre, the offset growth causes the increase in the neck-liner contact area. These curves show a rotation angle corresponding to the impingement, which is equal to 28.8° and 30.7° for each offset (0 mm and7 mm), respectively.

## Discussion

The stability of a hip in THA is assessed during operation in deep anaesthesia; thus, passive muscle reaction is predominant. To our best knowledge, no data are available in the literature concerning passive hip rotation preceded by flexion. Therefore, our validation was based only on experiments performed for passive flexion.

The observed agreement between the results produced by the model and experiments shown in Fig 4 is excellent. During this test, depending on the flexion angle, the tensile reaction of the joint is, in general assumed, to be carried by the extensors and flexors. In the intraoperative test, flexion is followed by internal rotation, and it is reasonable to perform additional examinations of the external rotators that are stretched during the second manoeuvre. Exploiting the limited experimental data, we extended our validation only by assessing the lengthening of the quadratus femoris and obturator externus muscles during flexion. This experiment has been performed on cadavers in which the muscle strings were simulated by wires [26].

The obtained numerical curves in Figs 5 and 6 approximate the experimental results sufficiently. It must be emphasized, the obturator externus, obturator internus, gemmelli and quadratus femoris are deep external rotators [12]. These muscles, together with the gluteus minimus, comprise the so-called ‘rotator cuff’ and because of their specific PCSA to fibre length ratio and characteristic location in the joint may be considered stabilizers of the hip, see [12]. However, little is known about the specific impact on the stabilization of each of these muscles.

Fig 8 demonstrates that the passive internal rotation moment is produced by the quadratus femoris and gluteus medius. However, the role of the former is much more significant in the deep rotation, which is manifested as the growth of the passive moment with the increasing internal rotation angle. The gluteus medius retains its neutral position during this manoeuvre; therefore, its passive rotation moment fades with deepening rotation.

A high offset increases the stretching of this muscle and provides the growth of its initial passive force because of the specific location of the quadratus femoris in the pelvis. The muscle is very short, runs horizontally in the neutral position and is the lowest muscle among the other deep rotators. Consequently, during flexion, the muscle undergoes large elongation—at 90° hip flexion, it extends by half of its optimal length (Fig 5). Thus, according to the Hill’s model, before starting the rotation, the passive force reaches approximately the value of the maximum isometric force, increasing the subsequent manoeuvre. Therefore, this muscle serves as a stiff band, providing stabilisation of the hip prosthesis, measured during the simulated intraoperative test.

**Fig 11:**
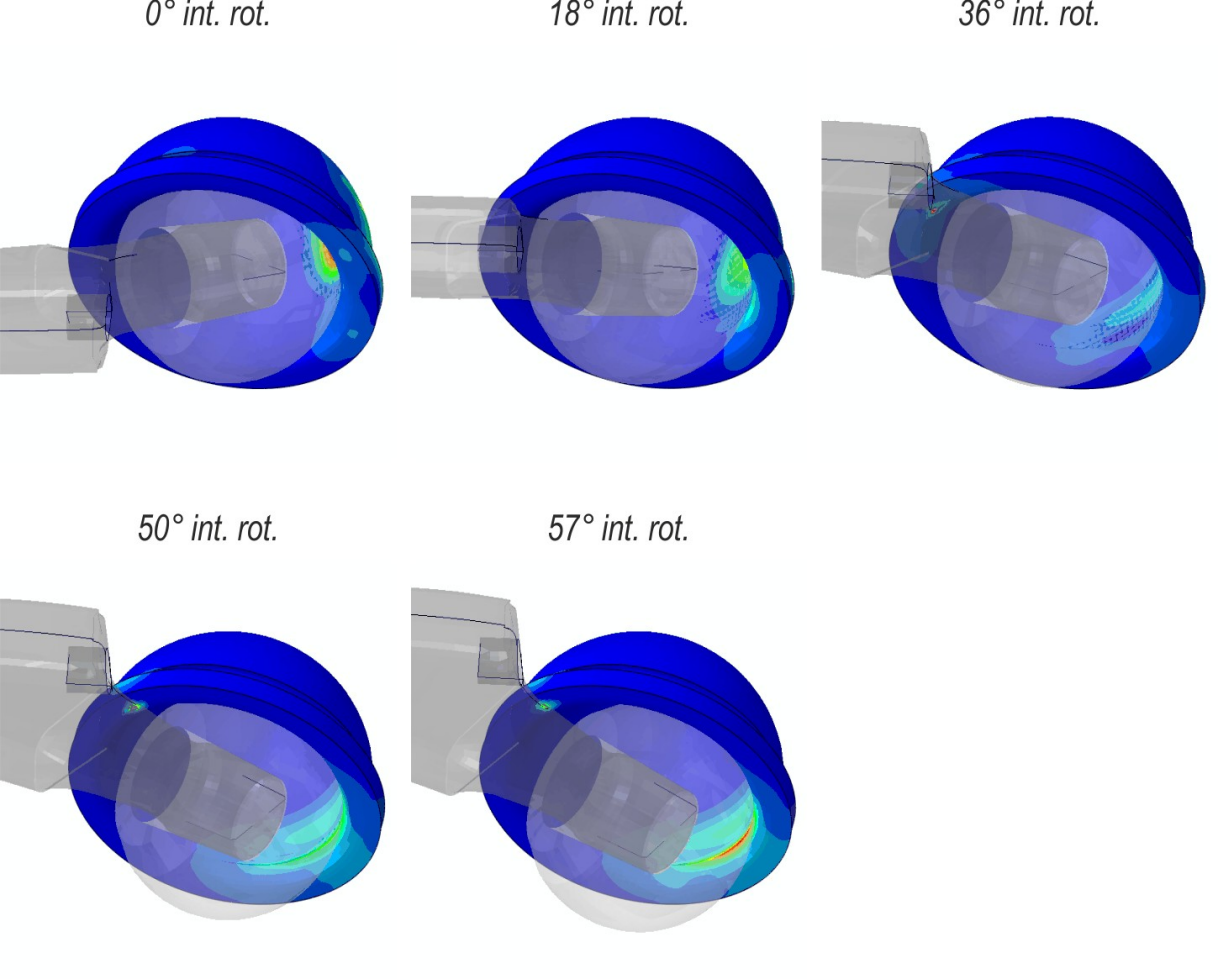
Lateral view of the liner during internal rotation.

Fig 10 indicates that, regardless of the offset value, the prosthetic head reaches the same dislocation displacement (≈5.5 mm) at the end of the analysis. At this moment, the analysed contact areas decrease significantly compared with their maximum values. The results shown in Fig 10 are supplemented by Fig 11, which presents the evolution of the Huber-Mises-Hencky (Mises stress) field in the liner for the offset at 0 mm. At the end of the analysis, the dislocation is noticed.

The study indicates the muscle which strengthening could potentially decrease the risk of the posterior dislocation of the hip prosthesis. The study of the problem will be extended in the future works.

This study possesses limitations. The muscles were modelled as nonlinear elastic springs acting in the anatomical direction of the given muscle. The three-dimensional structure of the muscle is not considered; thus, it is impossible to include mutual interactions between the muscles and fascia. Additionally, the muscle does not have transverse stiffness. The pelvis and femur dimensions for a single woman were used, whereas the properties of the muscles were considered as the means, independent of gender.

## Conclusions

In this work, we have shown for the first time that, during the popular hip dislocation test in the intraoperative manoeuvre from among of all the muscles crossing the hip joint, two muscles, the gluteus medius and quadratus femoris, produce the largest resisting moment opposing the external load exerted by the surgeon. With the increase in the internal rotation, however, the role of the gluteus medius ceases to be relevant. Thus, the quadratus femoris becomes the main soft tissue carrying the load applied to the joint. The increased offset value yields the increased passive muscle force in this muscle. We have shown that the moment causing hip dislocation may be interpreted as the limit point on the internal rotation moment curve.

Future studies should address the analysis of active muscles and their influence on the stability during everyday activities.

## Supporting information

S1 Appendix (DOCX)
