# Supplementary material for: Influence of the femoral offset on the muscles passive resistance in total hip arthroplasty
Source: PLoS One. 2021 May 4;16(5):e0250397. doi: 10.1371/journal.pone.0250397 (PMC8096111; doi:10.1371/journal.pone.0250397)
Supplement: S1 Appendix — (DOCX) [file pone.0250397.s001.docx]

**S1 Appendix**

The theoretical scheme of the musculo-tendon is illustrated in Fig. A1 The muscle and tendon are treated as springs attached in series. Both tissues resist if subjected to stretching. However, the muscle can also contract if it is activated by the motoneurons. Therefore, the tendon can be modelled as a single spring, whereas the muscle comprises two elements responsible for contraction and tensile resistance, working in parallel.

Fig. A1 Musculo-tendon theoretical scheme





From the series connection of the muscle and tendon, it follows that:

$F^{MT}=F^{M}\cdot\cos\alpha_{p}=F^{T}$,

where $F^{MT}$, $F^{M}$, and$F^{T}$represent forces in the musculo-tendon, muscle and tendon, respectively, and $\alpha_{p}$ is the muscle pennation angle.

According to the parallel connection of elements representing the muscle, the final force generated by this tissue is [1, 21, 24]:

$F^{M}=\left(F_{CE}^{M}+F_{PE}^{M}\right)$,

where $F_{CE}^{M}$ is the active force generated by the contractile element, and $F_{PE}^{M}$ is the muscle passive resistance.

The force induced in the contractile element is the so-called active force that can be obtained from [1, 2]:

$F_{CE}^{M}=F_{max}^{0}\cdot a(t)\cdot f_{CE}(L)\cdot f_{CE}(v)$.

$F_{max}^{0}$ is the maximum isometric (optimal) force of the muscle, $a(t)$represents the muscle activation level, $a(t)\in\left\langle0,1\right\rangle$, *L* is the muscle length (Fig. 1), *v* is the muscle contraction velocity,$f_{CE}(L)$ and $f_{CE}(v)$denote the force-length and force-velocity relationships, respectively [2, 23]. Because the intraoperative test in this study is simulated, the active force is generally negligible. The activation simulates only the muscle tone, which is assumed to be present to a very small extent also under the surgery conditions. Therefore, in our study, this parameter is considered a small and constant value (*a* =0.01). Additionally, because the analysed manoeuvres of the hip joint are presumed to be quasi-static, the force-velocity function is also constant and equal—i.e.,$f_{CE}(v)=1$(Fig. 1d). Thus, the force in the contractile element is only muscle length dependent. Using the force-length function $f_{CE}(L)$ proposed in [20], the$F_{CE}^{M}$value can be obtained from the following expression:

$F_{CE}^{M}=F_{max}^{0}\cdot a\cdot\exp\left(\frac{-(L^{*}-1)^{2}}{\gamma}\right)$ ,

where$L^{*}=L/L_{0}$is the normalised muscle length, $L_{0}$ is the optimal muscle fibre length, and $\gamma$is the curve shape parameter. According to [20],$\gamma=0.45$.

The muscle passive force is evaluated from the following general relationship [2]:

$F_{PE}^{M}=F_{max}^{0}\cdot f_{PE}(L)$ ,

where $f_{PE}(L)$represents the passive force-length relationship. Utilizing the proposal from [20], the value $F_{PE}^{M}$ follows from:

$F_{PE}^{M}=\left\{\begin{matrix} 0 & L^{*}<1 \\ F_{max}^{0}\cdot\frac{\exp\left(k_{sh}(L^{*}-1)/\varepsilon_{max}\right)-1}{\exp\left(k_{sh}\right)-1} & L^{*}\geq1 \end{matrix}\right.$ .

In (6) $k_{sh}$ is the curve shape parameter and $\varepsilon_{max}$is the muscle strain corresponding to the maximum isometric force. The present study assumes that$\varepsilon_{max}=0.7$ [3]. The shape parameter is usually set to a natural number from the range$\left\langle3,5\right\rangle$ [1, 20]. In the present study,$k_{sh}=4.$

The tendon is a typical passive element. The force-length expression for this tissue is considered a parabolic function after [4]:

$F^{T}=\left\{\begin{matrix} 0 & L_{T}<L_{slack} \\ \frac{F_{max}^{0}}{(\varepsilon_{max,T}\cdot L_{slack})^{2}}\left(L_{T}-L_{slack}\right)^{2} & L_{T}\geq L_{slack} \end{matrix}\right.$ ,

where $L_{T}$and$L_{slack}$ are the tendon and tendon slack lengths, respectively, and $\varepsilon_{\max,T}$ is the tendon strain corresponding to the maximum isometric force. In the present study, similar to [4],$\varepsilon_{max,T}=0.04$for all muscles.

The material data of the muscles and tendons crossing the hip joint is taken from [22, 25] and presented in Table 1.

Table 1 Muscle and tendon parameters

| Full name | Optimal force [N] | Optimal fibre length [mm] | Tendon slack length [mm] | Pennation angle [°] |
| --- | --- | --- | --- | --- |
| Adductor brevis | 303.70 | 103.00 | 36.53 | 6.10 |
| Adductor longus | 399.50 | 108.00 | 124.67 | 7.10 |
| Adductor magnus distal | 324.20 | 177.00 | 75.20 | 13.80 |
| Adductor magnus ischial | 324.20 | 156.00 | 175.47 | 11.90 |
| Adductor magnus middle | 324.20 | 138.00 | 17.09 | 14.70 |
| Adductor magnus proximal | 324.20 | 106.00 | 10.39 | 22.20 |
| Biceps femoris long head | 705.20 | 98.00 | 262.49 | 11.60 |
| Gemelli superior | 41.48 | 48.50 | 13.40 | 0.00 |
| Gemelli inferior | 62.20 | 55.60 | 0.00 | 1.02 |
| Gluteus maximus superior | 546.10 | 147.00 | 37.18 | 21.10 |
| Gluteus maximus middle | 780.50 | 157.00 | 52.32 | 21.90 |
| Gluteus maximus inferior | 526.10 | 167.00 | 77.83 | 22.80 |
| Gluteus medius anterior | 881.10 | 73.00 | 52.26 | 20.50 |
| Gluteus medius middle | 616.50 | 73.00 | 51.73 | 20.50 |
| Gluteus medius posterior | 702.00 | 73.00 | 47.05 | 20.50 |
| Gluteus minimus anterior | 180.00 | 68.00 | 15.71 | 10.00 |
| Gluteus minimus middle | 190.00 | 56.00 | 37.06 | 0.00 |
| Gluteus minimus posterior | 215.00 | 38.00 | 67.34 | 1.00 |
| Gracilis | 137.30 | 228.00 | 124.95 | 8.20 |
| Iliacus | 621.90 | 107.00 | 120.00 | 14.30 |
| Obturator Externus | 276.94 | 80.40 | 5.90 | 1.67 |
| Obturator Internus | 229.36 | 87.70 | 35.10 | 2.50 |
| Pectineus | 177.00 | 132.00 | 1.74 | 0.00 |
| Piriformis | 130.20 | 83.70 | 60.35 | 2.80 |
| Psoas | 479.70 | 117.00 | 192.41 | 10.70 |
| Quadratus femoris | 228.80 | 63.70 | 0 | 0.00 |
| Rectus femoris | 848.80 | 76.00 | 367.31 | 13.90 |
| Sartorius | 113.50 | 403.00 | 60.76 | 1.30 |
| Semimembranosus | 162.70 | 69.00 | 291.83 | 15.10 |
| Semitendinosus | 301.90 | 193.00 | 166.13 | 12.90 |
| Tensor fascia latae | 155.00 | 149.00 | 0 | 3.00 |

**References**

1. Romero F, Alonso FJ. A comparison among different Hill-type contraction dynamics formulations for muscle force estimation. Mech Sci. 2016;7: 19–29. doi:10.5194/ms-7-19-2016

2. Millard M, Uchida T, Seth A, Delp SL. Flexing computational muscle: Modeling and simulation of musculotendon dynamics. J Biomech Eng. 2013;135: 1–11. doi:10.1115/1.4023390

3. Catelli DS, Wesseling M, Jonkers I, Lamontagne M. A musculoskeletal model customized for squatting task. Comput Methods Biomech Biomed Engin. 2019;22: 21–24. doi:10.1080/10255842.2018.1523396

4. van Soest AJ, Bobbert MF. The contribution of muscle properties in the control of explosive movements. Biol Cybern. 1993;69: 195–204. doi:10.1007/BF00198959
